# Association of transcobalamin c. 776C>G with overall survival in patients with primary central nervous system lymphoma

**DOI:** 10.1038/bjc.2012.476

**Published:** 2012-10-25

**Authors:** M Linnebank, S Moskau, A Kowoll, A Semmler, C Bangard, M Vogt-Schaden, G Egerer, G Schackert, H Reichmann, I G H Schmidt-Wolf, H Pels, U Schlegel

**Affiliations:** 1 Department Neurology, University Hospital Bonn, Bonn, Germany; 2 Department Neurology, University Hospital Zurich, Frauenklinikstrasse 26, Zurich CH-8091, Switzerland; 3 Department Neurology, University Hospital Bochum, Knappschaftskrankenhaus, Bochum, Germany; 4 Department Radiology, University Hospital Cologne, Cologne, Germany; 5 Department Neurology, University Hospital Heidelberg, Heidelberg, Germany; 6 Department Medicine, Haemato-Oncology, University Hospital Heidelberg, Heidelberg, Germany; 7 Department Neurosurgery, University Hospital Dresden, Dresden, Germany; 8 Department Neurology, University Hospital Dresden, Dresden, Germany; 9 Department Medicine III, University Hospital Bonn, Bonn, Germany; 10 Department Neurology, Krankenhaus Barmherzige Brüder, Regensburg, Germany

**Keywords:** primary central nervous system lymphoma, polymorphism, survival

## Abstract

**Background:**

Chemotherapy for primary central nervous system lymphoma (PCNSL) is based on methotrexate (MTX), which interferes with both nucleic acid synthesis and methionine metabolism. We have reported previously that genetic variants with influence on methionine metabolism are associated with MTX side effects, that is, the occurrence of white matter lesions as a sign of MTX neurotoxicity. Here, we investigated whether such variants are associated with MTX efficacy in terms of overall survival in MTX-treated PCNSL patients.

**Methods:**

We analysed seven genetic variants influencing methionine metabolism in 68 PCNSL patients treated with systemic and facultative intraventricular MTX-based polychemotherapy (Bonn protocol).

**Results:**

Median age at diagnosis was 59 years (range: 28–77), 32 patients were female. Younger age (Wald=8.9; *P*=0.003) and the wild-type C (CC) allele of the genotype transcobalamin c (Tc2). 776C>G (Wald=6.7; *P*=0.010) were associated with longer overall survival in a multivariate COX regression analysis.

**Conclusion:**

This observation suggests that the missense variant Tc2. 776C>G influences both neurotoxicity and efficacy of MTX in the Bonn PCNSL protocol.

Primary central nervous system lymphomas (PCNSL) represent ∼2.4% of all primary brain tumours in the United States (Central Brain Tumour Registry of the United States, 2004–2007). Untreated PCNSL has a poor prognosis of a median survival of only a few weeks. Treatment protocols include chemotherapies and brain irradiation. All first-line chemotherapy protocols in PCNSL are based on high-dose methotrexate (MTX), a competitive inhibitor of dihydrofolate reductase (DHFR), which synthesizes 5,10-methylenetetrahydrofolate (5,10-MTHF; Figure 1 (Finkelstein, 1990). Thereby, MTX inhibits nucleic acid synthesis. However, 5,10-MTHF is also used for the synthesis of 5-methyltetrahydrofolate (5-MTHF) by 5,10-MTHF reductase (MTHFR). Together with vitamin B12, which is transported by transcobalamin 2 (Tc2), 5-MTHF serves as cofactor of methionine synthase, which synthesizes methionine from homocysteine. Methionine can be activated to S-adenosylmethionine, which acts as a methyl group donor, for example, for CNS myelination. The reduced folate carrier 1 (RFC1) is involved in the uptake of folate and MTX into the CNS.

Variants of genes encoding these enzymes, transporter and carrier proteins of methionine metabolism are associated with MTX neurotoxicity as indicated by CNS white matter hyperintensities in T2-weighted magnetic resonance imaging (MRI) in PCNSL patients (Linnebank *et al*, 2005, 2007, 2009). In this study, we aimed at investigating whether these variants are also associated with the overall survival of PCNSL patients.

## Methods

We analysed 68 consecutive PCNSL patients treated with the Bonn protocol with (*n*=42) or without (*n*=26) intraventricular therapy (Pels *et al*, 2003, 2009). Median age at diagnosis was 59 years (range: 28–77), 32 patients were female. Median Karnofsky score was 70 (range: 50–100). Median time of follow-up was 44 months (range: 1–114). The MTX-based Bonn protocol with and without intraventricular treatment has been described in detail (Pels *et al*, 2003, 2009). In brief, the protocol consisted of six chemotherapy cycles with intravenous high-dose MTX (cycles 1, 2, 4 and 5), high-dose cytarabine (ara-C; cycles 3 and 6) and vinca-alkaloids, oral dexamethasone and (until 2002) intraventricular MTX, ara-C and prednisolone. Whereas 62 patients received the complete protocol, 6 patients terminated early because of progressive disease. In the entire population of patients intended to be treated, we analysed seven genetic variants influencing methionine metabolism by PCR amplification of genomic DNA prepared from peripheral leucocytes and subsequent endonuclease restriction, where applicable. Amplicons were analysed by agarose gel elecectrophoresis as published: CBS 844ins68, DHFR c.594+59del19bp, MTHFR c.677C>T, MTHFR c.1298A>C, MTR c.2756A>G, RFC1 c.80G>A, Tc2 c.776C>G (Linnebank *et al*, 2005, 2007, 2009). Multivariate COX regression analysis (forward stepwise; Wald) was used to analyse the association of age, gender, Karnofsky score before treatment, type of Bonn protocol (with or without intraventricular therapy) and the seven variants of methionine metabolism with overall survival as the primary parameter of interest. In addition, the association of the genetic variants with further dependent variables was tested in exploratory analyses, together with the same co-variables as before: treatment response (complete remission (CR), including CR unconfirmed, *vs* incomplete response/others; multinominal regression analysis), response duration (COX regression), time to treatment failure (COX regression) and Karnofsky performance score (analysis of variance (ANOVA); Pels *et al*, 2003, 2009). Overall survival was determined from time of histological diagnosis to date of death. Response duration was calculated from the time when response was first documented to relapse. Time to treatment failure was calculated from initiation of treatment to date of disease progression, relapse, death or premature termination of therapy due to any cause (Pels *et al*, 2003). Response was categorised according to a consensus of the International PCNSL cooperative group (Abrey *et al*, 2005). Median overall survival was not reached at the time of this analysis; thus, we provide mean values where applicable. Deviations of genotype distributions from the Hardy–Weinberg equation were tested by a *χ*^*2*^ goodness-of-fit test. Univariate log rank analyses with Kaplan–Meier curves were used to illustrate data. Analysis of variance was used to exclude significant differences of age between groups defined by Tc2 genotypes. Threshold was defined with *α*<0.05. All patients or their legal trustees gave informed written consent to this study, which was approved by the Local Ethics Committee.

## Results

Genotyping succeeded for all patients. No genotype distribution deviated from the Hardy–Weinberg equation. In multivariate COX analysis of the intent-to-treat-population, younger age was significantly associated with longer survival (Wald=6.4; *P*=0.011), but not clearly with higher Karnofsky performance score before therapy (Wald=2.7; *P*=0.098). Gender showed no significant association in this population (Wald=0.21; *P*=0.649). In addition, the wild-type C allele of Tc2 c.776C>G was associated with overall survival, suggesting a gene-dose effect (Table 1; Figure 2). Of the group of patients with the CC genotype, two patients died due to tumour progression. Of the group with the CG genotype, four patients died due to tumour progression, one patient due to therapy-associated toxicity and three patients probably due to tumour progression, which was not proved by imaging. Of the group with the GG genotype, five patients died due to tumour progression. Of those six patients who did not complete treatment due to early progression, each two carried the genotypes CC, CG and GG not suggesting that the Tc2 genotype was associated with incomplete treatment.

In explorative secondary analyses, patients with the wild-type CC genotype had a longer response duration (median not yet reached; mean: 74±11 months) and a longer time to treatment failure (61±12 months) than the other patients (43±9 and 38±7, respectively; not significant). Further, 14 of the 20 patients (0.70) homozygous for the wild-type CC genotype of Tc2 c.776C>G had a CR compared with only 24 of the 48 patients (0.50) with the mutant CG or GG genotypes, but this was not significant, either. In addition, the wild-type C allele was associated with a better Karnofsky performance score after treatment for trend (*P*=0.054; Table 2). None of the patients who completed therapy suffered from overt neurotoxicity. However, several patients developed white matter changes visible in T2 FLAIR MRI as published previously. If development of such white matter changes was set as covariable in the COX analysis, the association of Tc2 c.776C>G with overall survival was still significant (*P*=0.03), whereas the other genotypes still did not show significant associations (not shown). The patient groups defined by Tc2 genotypes did not differ in age, CC (56±11 years), CG (58±12) and GG (56±14; *P*=0.722).

## Discussion

Current MTX-based treatment protocols for PCNSL achieve high response rates and clearly have improved prognosis. However, a substantial fraction of patients experience serious adverse events during treatment, have incomplete responses only or suffer from relapses. Individual factors like age and Karnofsky performance score before treatment are predictive concerning the clinical outcome (Ferreri *et al*, 2003; Abrey *et al*, 2006; Jahnke and Thiel, 2009). This was confirmed in our study (Karnofsky performance score: trend only) arguing for the representativity of the study population. Another predictive factor, that is, early complete response to initial treatment (Pels *et al*, 2010) was not analysed in this study. The identification of additional factors like genetic variants may help to further improve individual prediction of outcome for different therapeutic options.

In this study, we report that the mutant G allele of Tc2 c.776C>G was associated with shorter overall survival of PCNSL patients treated with the high-dose MTX-based Bonn protocol (Table 1). We have reported previously that this mutant G allele is associated with MTX neurotoxicity as indicated by white matter changes detected in T2-weighted magnet resonance imaging MRI (Linnebank *et al*, 2005, 2009). Interestingly, in this study, we also observed an association of this mutant variant with lower Karnofsky performance scores after therapy for trend as a further possible indicator of the relevance of this variant for MTX toxicity. Alternatively, the lower Karnofsky performance score in carriers of the G-allele may also be explained by reduced therapeutic efficacy of MTX as suggested by the data summarised in Table 2.

**Figure 1:**
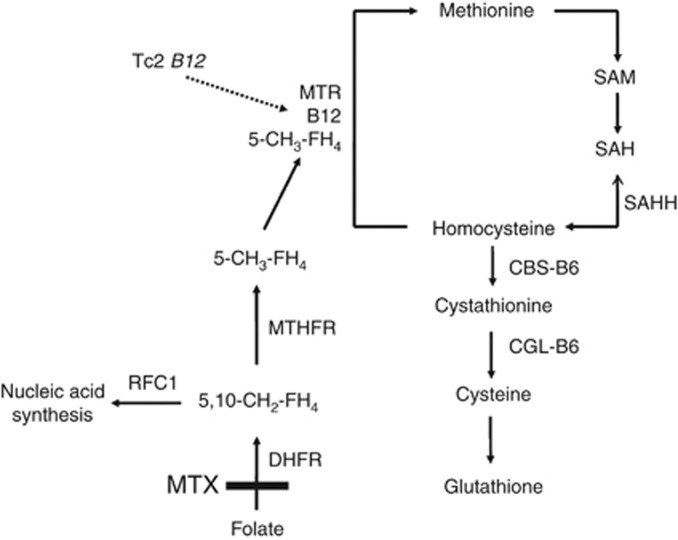
Human methionine metabolism. Methotrexate (MTX) inhibits DHFR, which synthesizes 5,10-CH_2_-FH_4_ from the folate pool in two subsequent steps. The RFC1 is involved in the uptake of 5,10-CH_2_-FH_4_ and MTX to tissues and organs including the CNS. 5,10-CH_2_-FH_4_ is needed for nucleic acid synthesis and, alternatively, can be reduced by MTHFR to 5-MTHF (5-CH_3_-FH_4_). Together with methylcobalamin, 5-CH_3_-FH_4_ is used by 5-MTHF-homocysteine S-methyltransferase (MTR, also called methionine synthase) to synthesise methionine. Transcobalamin 2 (Tc2) is the major transporter protein for cobalamin. Activated methionine (S-adenosylmethionine, SAM) is the methyl group donor for numerous reactions. The degradation product of SAM is S-adenosylhomocysteine (SAH), which is hydrolysed to homocysteine by SAH-hydrolase. Homocysteine can be transsulfurated to cystathionine by the vitamin B6-dependent cystathionine beta-synthase (CBS), and subsequently, via the vitamin B6-dependent cystathionine gamma-lyase (CGL), to cysteine. Cysteine is essential for glutathione synthesis. (Finkelstein, 1990).

**Figure 2:**
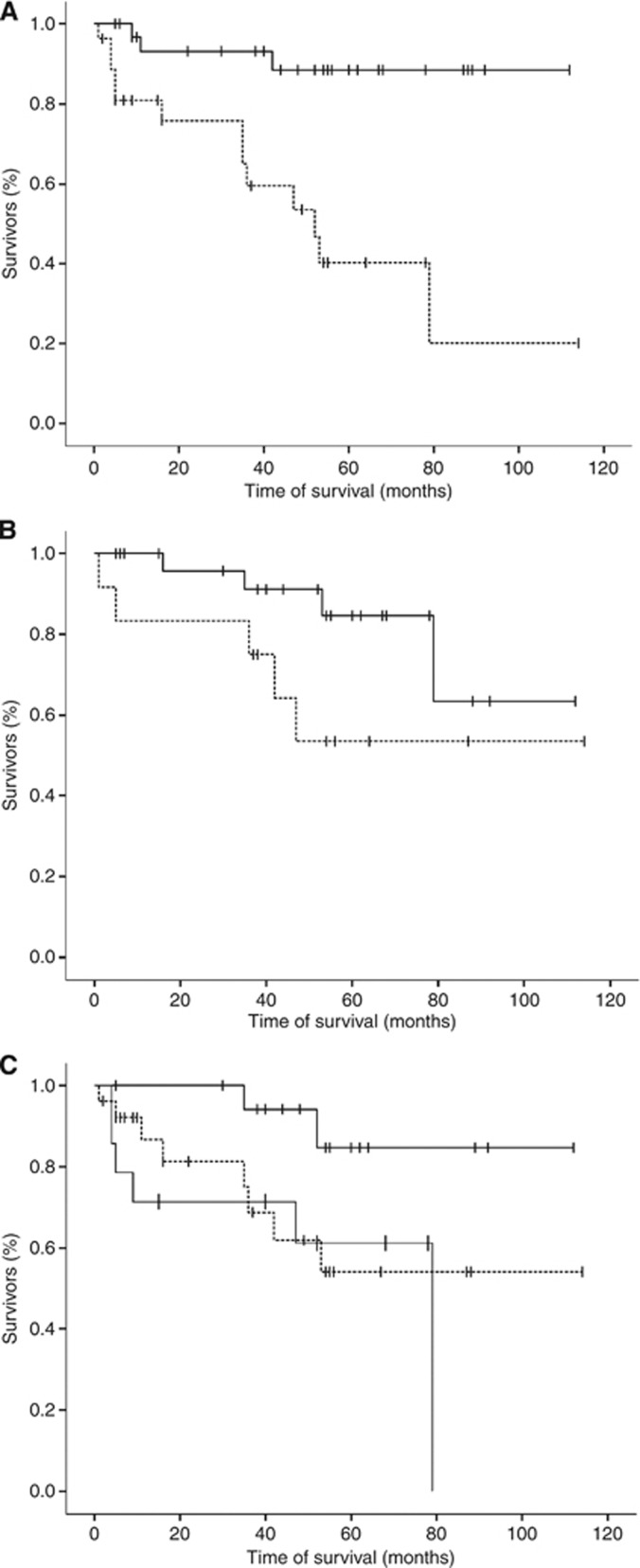
Age, Kanrofsky performance score, Tc2 c.776C>G and overall survival. Overall survival of patients grouped by (**A**) age (<60=black line, ⩾60 years=broken line), (**B**) Karnofsky performance score (<70=broken line, ⩾70=black line) and (**C**) Tc2 c.776C>G genotype (CC=black line, CG=broken line, GG=grey line) is illustrated by Kaplan–Meier curves. Censored cases are indicated by upright dashes. *x* axis: months; *y* axis: proportion of patients alive.

Transcobalamin c is the transporter protein of cobalamin (vitamin B12), which is necessary to remethylate methionine and S-adenosylmethionine from homocysteine. S-adenosylmethionine is needed to synthesise, for example, myelin basic protein, phospholipids and sphingomyelins. Such molecules are necessary for CNS integrity and myelination, and, accordingly, deficiency of S-adenosylmethionine can cause demyelination of the CNS (Figure 1; Surtees *et al*, 1991). The missense mutant G variant of Tc2 c.776C>G (p.P259R) lowers the affinity of Tc2 to cobalamin and leads to reduced concentrations of blood Tc2–cobalamin complexes, for example, reducing the biological availability of cobalamin for methionine and S-adenosylmethionine synthesis (von Castel-Dunwoody *et al*, 2005). This may explain why the mutant G-allele of Tc2 c.776C>G is associated with progressive demyelination in patients with X-chromosomal adrenoleukodystrophy and the occurrence of white matter T2 hyperintensities on brain MRI in PCNSL patients treated with the MTX-based Bonn protocol (Linnebank *et al*, 2005, 2009; Semmler *et al*, 2009). During MTX treatment, patients with one or two mutant G alleles and, as consequence, with a lower availability of cobalamin for 5-MTHF-dependent methionine synthesis will experience pronounced MTX-induced S-adenosylmethionine depletion leading to a higher risk of neurotoxicity and a lower consumption of 5,10-MTHF for 5-MTHF synthesis (Figure 1). In parallel, more 5,10-MTHF might be left for nucleic acid synthesis, decreasing MTX efficacy as one possible explanation of an association with overall survival observed in this cohort (Figure 2c). In our study, additional explorative analyses, which were not powered to yield significant results with respect to the small subgroup sizes, suggested that the association of Tc2 c.776C>G with overall survival may be explained by both, a modification of instantaneous MTX treatment efficacy and toxicity. This is indicated by the post treatment Karnofsky performance score and by sustained treatment efficacy, as indicated by the time to relapse and time to treatment failure. As limitations, the results of our study remain to be retested in an independent population of PCNSL patients and, preferably, also other MTX-treated populations. We cannot exclude that other factors like differences in the MTX concentrations in the target tissue have confounded our findings. Additional mechanisms including an interaction between Tc2 c.776C>G and rescue with leucovorin (formyltetrahydrofolate), which is converted to 5,10-MTHF antagonising MTX, may also be relevant, but were not analysed in our study. Further, the biological consequences of Tc2 c.776C>G may be modified by vitamin B12 or folate plasma levels (Stanislawska-Sachadyn *et al*, 2010), which were not measured in our study population.

## Figures and Tables

**Table 1:** Variants and mean overall survival (months)

**Variant**	**Genotype and overall survival**			**COX-regression: Wald;** ***P***
CBS844ins68	del/del (0.82)	ins/del (0.18)	ins/ins (−)	
	81±7	56±9	Not observed	1.1; 0.304
DHFR c.594+59del19bp	del/del (0.20)	ins/del (0.50)	ins/ins (0.31)	
	87±13	78±10	60±9	2; 0.162
MTHFR c.677C>T	CC (0.52)	CT (0.36)	TT (0.13)	
	76±10	64±8	101±12	1; 0.310
MTHFR c.1298 A>C	AA (0.53)	AC (0.37)	CC (0.10)	
	81±9	75±11	60±11	0.018; 0.892
MTR c.2756A>G	AA (0.66)	AG/GG (0.34)		
	79±8	79±11		0.38; 0.537
RFC1 c.80G>A	GG (0.31)	AG (0.42)	AA (0.26)	
	78±12	72±11	68±5	0.69; 0.408
Tc2 c.776C>G	CC (0.34)	CG (0.44)	GG (0.23)	
	100±8	76±11	52±10	7.3; 0.007

Abbreviations: CBS=cystathionine beta-synthase; DHFR=dihydrofolate reductase; MTR=5-methyltetrahydrofolate-homocysteine S-methyltransferase; MTHFR=MTHF reductase; RFC1=reduced folate carrier 1; Tc2=transcobalamin 2.

Median overall survival was not yet reached. Thus, mean overall survival in months±1 s.d. is depicted. *P*-values refer to multivariate analysis with age and gender and Karnofsky performance score before therapy as co-variables. The GG genotype of MTR c.2756A>G was only observed in two patients. Thus, for statistical analysis, GG was pooled with AG.

**Table 2:** Tc2. 776C>G genotypes and mean Karnofsky scores

**Tc2**	**CC**	**CG**	**GG**	**ANOVA: F;** ***P***
Karnofsky score before treatment	77±12	71±18	70±17	0.62; 0.543
Karnofsky score after treatment	92±7	81±25	67±38	3.16; 0.054

Abbreviations: ANOVA=analysis of variance; Tc2=transcobalamin c.

## References

[bib1] Abrey LE, Batchelor TT, Ferreri AJ, Gospodarowicz M, Pulczynski EJ, Zucca E, Smith JR, Korfel A, Soussain C, DeAngelis LM, Neuwelt EA, O'Neill BP, Thiel E, Shenkier T, Graus F, van den Bent M, Seymour JF, Poortmans P, Armitage JO, Cavalli F (2005) Report of an international workshop to standardize baseline evaluation and response criteria for primary CNS lymphoma. J Clin Oncol 23: 5034–5043 15955902 10.1200/JCO.2005.13.524

[bib2] Abrey LE, Ben-Porat L, Panageas KS, Yahalom J, Berkey B, Curran W, Schultz C, Leibel S, Nelson D, Mehta M, DeAngelis LM (2006) Primary central nervous system lymphoma: the Memorial Sloan-Kettering Cancer Center prognostic model. J Clin Oncol 24: 5711–5715 17116938 10.1200/JCO.2006.08.2941

[bib3] Ferreri AJ, Blay JY, Reni M, Pasini F, Spina M, Ambrosetti A, Calderoni A, Rossi A, Vavassori V, Conconi A, Devizzi L, Berger F, Ponzoni M, Borisch B, Tinguely M, Cerati M, Milani M, Orvieto E, Sanchez J, Chevreau C, DellOro S, Zucca E, Cavalli F (2003) Prognostic scoring system for primary CNS lymphomas: the International Extranodal Lymphoma Study Group experience. J Clin Oncol 21: 266–272 12525518 10.1200/JCO.2003.09.139

[bib4] Finkelstein JD (1990) Methionine metabolism in mammals. J Nutr Biochem 1: 228–237 15539209 10.1016/0955-2863(90)90070-2

[bib5] Jahnke K, Thiel E (2009) Treatment options for central nervous system lymphomas in immunocompetent patients. Expert Rev Neurother 9: 1497–1509 19831839 10.1586/ern.09.100

[bib6] Linnebank M, Malessa S, Moskau S, Semmler A, Pels H, Klockgether T, Schlegel U (2007) Acute methotrexate-induced encephalopathy – causal relation to homozygous allelic state for MTR c.2756A>G (D919G)? J Chemother 19: 455–457 17855192 10.1179/joc.2007.19.4.455

[bib7] Linnebank M, Moskau S, Jurgens A, Simon M, Semmler A, Orlopp K, Glasmacher A, Bangard C, Vogt-Schaden M, Urbach H, Schmidt-Wolf IG, Pels H, Schlegel U (2009) Association of genetic variants of methionine metabolism with methotrexate-induced CNS white matter changes in patients with primary CNS lymphoma. Neuro Oncol 11: 2–8 18806228 10.1215/15228517-2008-082 PMC2718955

[bib8] Linnebank M, Pels H, Kleczar N, Farmand S, Fliessbach K, Urbach H, Orlopp K, Klockgether T, Schmidt-Wolf IG, Schlegel U (2005) MTX-induced white matter changes are associated with polymorphisms of methionine metabolism. Neurology 64: 912–913 15753437 10.1212/01.WNL.0000152840.26156.74

[bib9] Pels H, Juergens A, Glasmacher A, Schulz H, Engert A, Linnebank M, Schackert G, Reichmann H, Kroschinsky F, Vogt-Schaden M, Egerer G, Bode U, Schaller C, Lamprecht M, Hau P, Deckert M, Fimmers R, Bangard C, Schmidt-Wolf IG, Schlegel U (2009) Early relapses in primary CNS lymphoma after response to polychemotherapy without intraventricular treatment: results of a phase II study. J Neurooncol 91: 299–305 18931887 10.1007/s11060-008-9712-4

[bib10] Pels H, Juergens A, Schirgens I, Glasmacher A, Schulz H, Engert A, Schackert G, Reichmann H, Kroschinsky F, Vogt-Schaden M, Egerer G, Bode U, Deckert M, Fimmers R, Urbach H, Schmidt-Wolf IG, Schlegel U (2010) Early complete response during chemotherapy predicts favorable outcome in patients with primary CNS lymphoma. Neuro Oncol 12: 720–724 20159882 10.1093/neuonc/noq010 PMC2940662

[bib11] Pels H, Schmidt-Wolf IG, Glasmacher A, Schulz H, Engert A, Diehl V, Zellner A, Schackert G, Reichmann H, Kroschinsky F, Vogt-Schaden M, Egerer G, Bode U, Schaller C, Deckert M, Fimmers R, Helmstaedter C, Atasoy A, Klockgether T, Schlegel U (2003) Primary central nervous system lymphoma: results of a pilot and phase II study of systemic and intraventricular chemotherapy with deferred radiotherapy. J Clin Oncol 21: 4489–4495 14597744 10.1200/JCO.2003.04.056

[bib12] Semmler A, Bao X, Cao G, Kohler W, Weller M, Aubourg P, Linnebank M (2009) Genetic variants of methionine metabolism and X-ALD phenotype generation: results of a new study sample. J Neurol 256(8): 1277–1280 19353223 10.1007/s00415-009-5114-6

[bib13] Stanislawska-Sachadyn A, Woodside JV, Sayers CM, Yarnell JW, Young IS, Evans AE, Mitchell LE, Whitehead AS (2010) The transcobalamin (TCN2) 776C>G polymorphism affects homocysteine concentrations among subjects with low vitamin B(12) status. Eur J Clin Nutr 64: 1338–1343 20808328 10.1038/ejcn.2010.157

[bib14] Surtees R, Leonard J, Austin S (1991) Association of demyelination with deficiency of cerebrospinal-fluid S-adenosylmethionine in inborn errors of methyl-transfer pathway. Lancet 338: 1550–1554 1683972 10.1016/0140-6736(91)92373-a

[bib15] von Castel-Dunwoody KM, Kauwell GP, Shelnutt KP, Vaughn JD, Griffin ER, Maneval DR, Theriaque DW, Bailey LB (2005) Transcobalamin 776C->G polymorphism negatively affects vitamin B-12 metabolism. Am J Clin Nutr 81: 1436–1441 15941899 10.1093/ajcn/81.6.1436

